# Supplementary figures and images for: Patterns of prescription dispensation and over-the-counter medication sales in Sweden during the COVID-19 pandemic
Source: PLoS One. 2021 Aug 13;16(8):e0253944. doi: 10.1371/journal.pone.0253944 (PMC8362980; doi:10.1371/journal.pone.0253944)

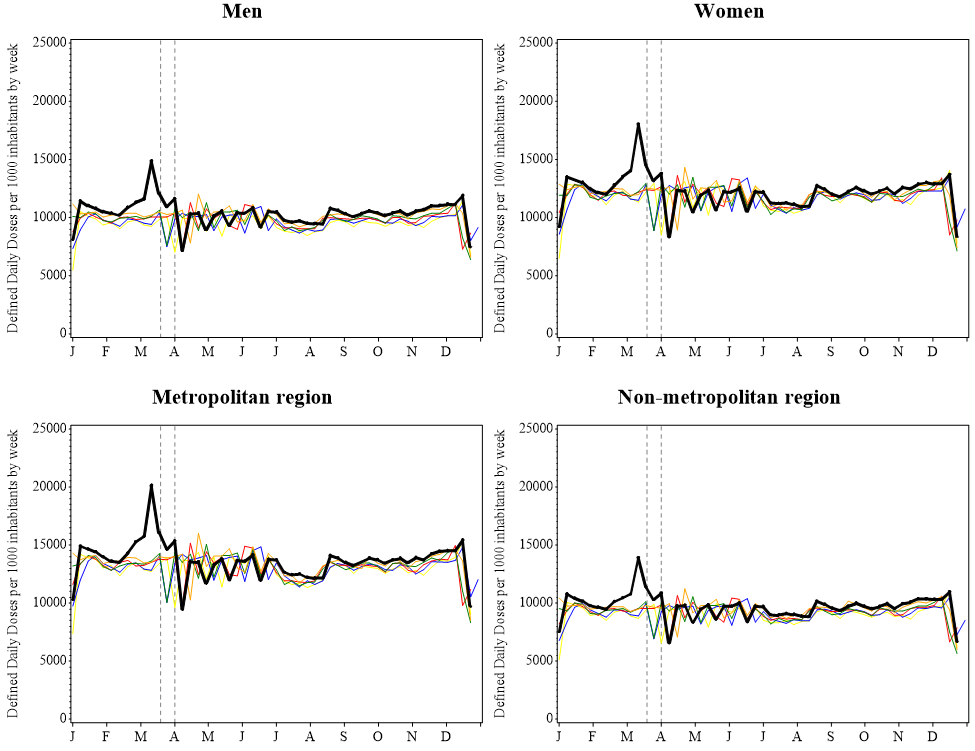

Supplement: S1 Fig — Note: The vertical lines indicate the weeks containing March 19th and April 1st 2020 when limits on medication sales were recommended and then mandated, respectively. (TIF) [file pone.0253944.s001.tif]

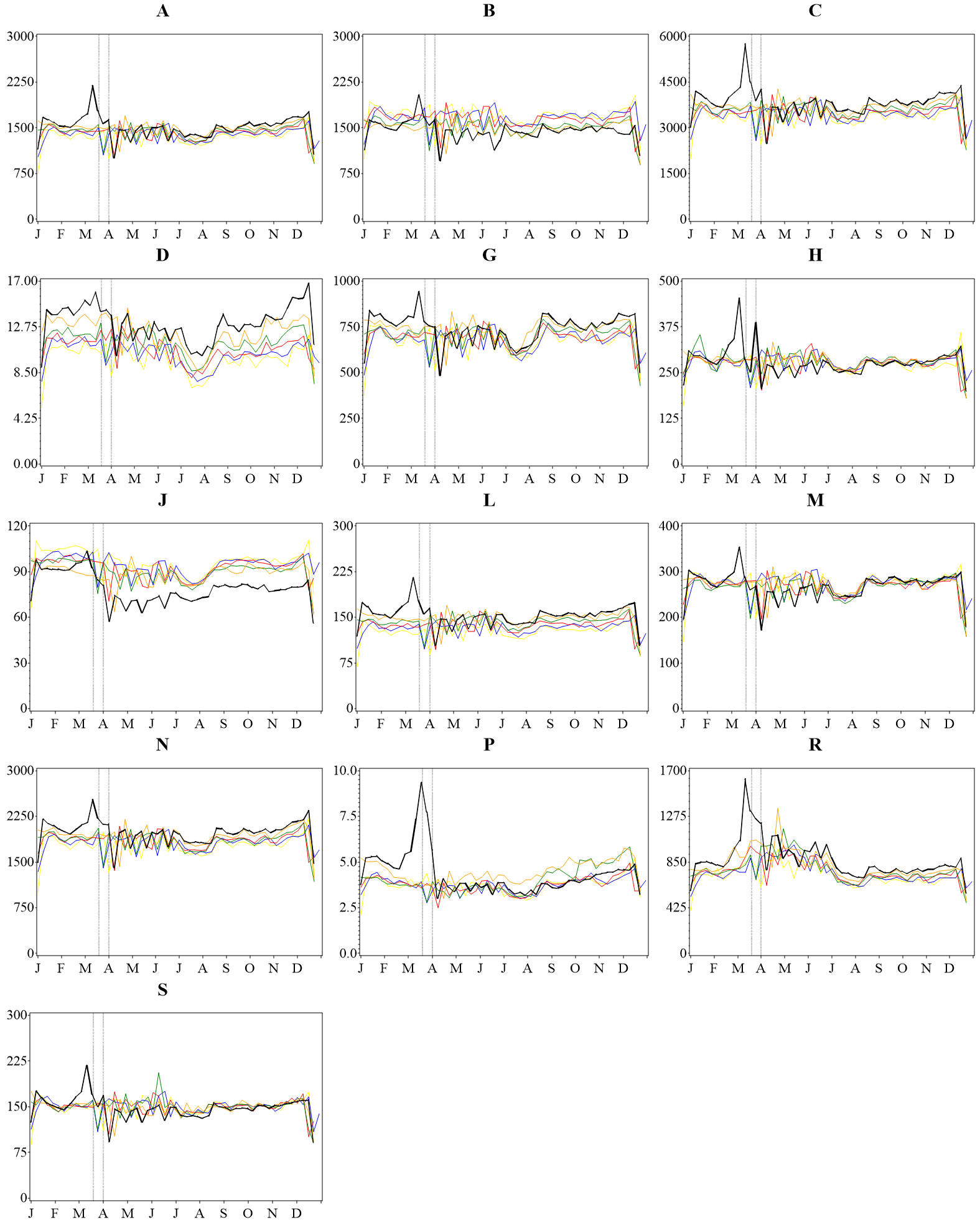

Supplement: S2 Fig — Note: The vertical lines indicate the weeks containing March 19th and April 1st 2020 when limits on medication sales were recommended and then mandated, respectively. (TIF) [file pone.0253944.s002.tif]

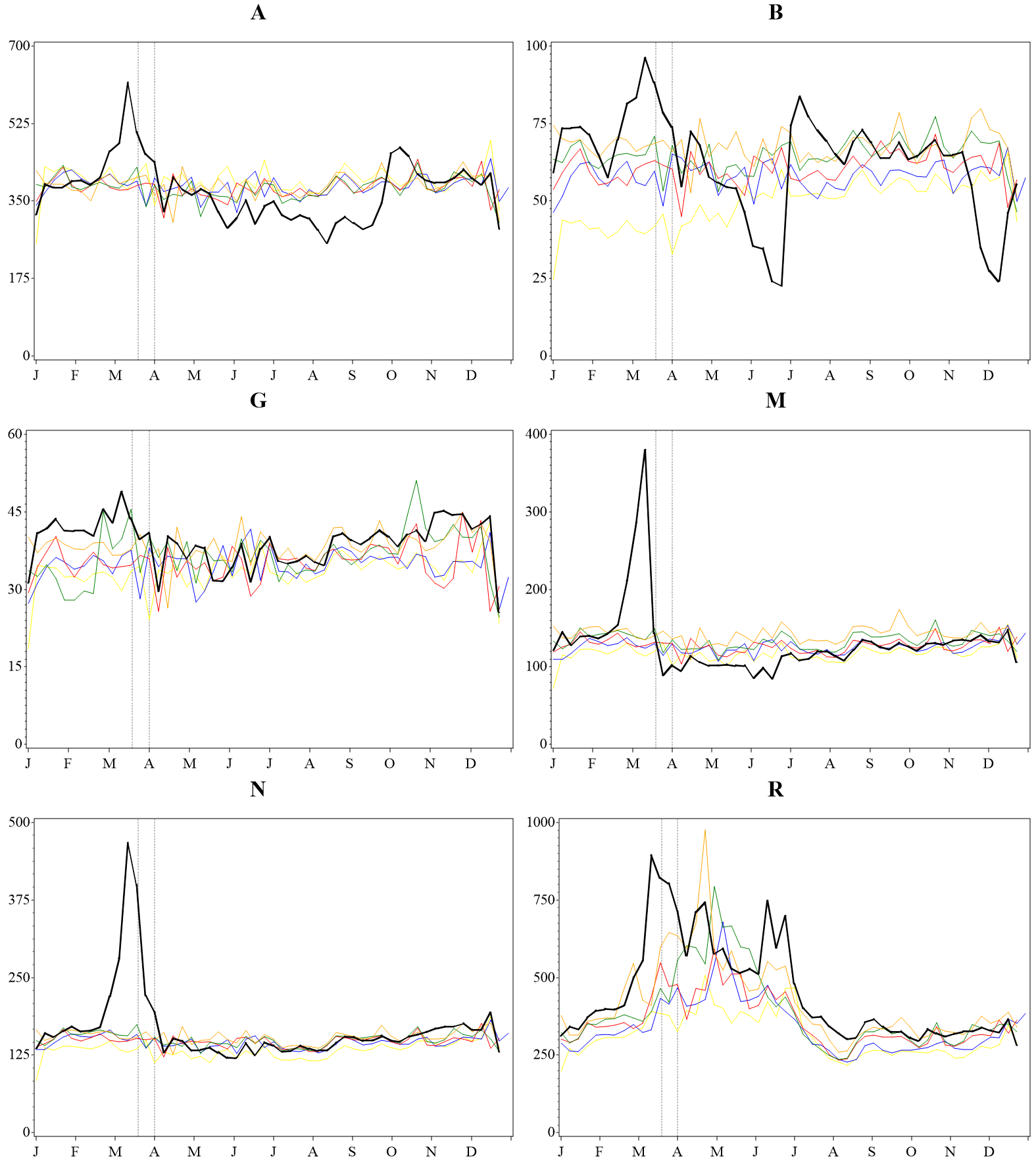

Supplement: S3 Fig — Note: The vertical lines indicate the weeks containing March 19th and April 1st 2020 when limits on medication sales were recommended and then mandated, respectively. (TIF) [file pone.0253944.s003.tif]
