# Supplementary material for: Patterns of prescription dispensation and over-the-counter medication sales in Sweden during the COVID-19 pandemic
Source: PLoS One. 2021 Aug 13;16(8):e0253944. doi: 10.1371/journal.pone.0253944 (PMC8362980; doi:10.1371/journal.pone.0253944)
Supplement: S1 Table — (PDF) [file pone.0253944.s004.pdf]

**Supplementary Table 1** Observed versus predicted volume of dispensed defined daily doses of prescription medication per 1000 inhabitants in Sweden for weeks in 2020 with statistically significant differences

| | number of DDD per 1000 | | | | | | | | |
|---|---|---|---|---|---|---|---|---|---|
| | ATC anatomical main group | Week start | Observed | Predicted | St Error | Lower 95% CL | Upper 95% CL | Ratio (observed/ predicted) | p-value* |
| | Any ATC | 2020-03-04 | 12809 | 11142 | 364 | 10424 | 11861 | 1.15 | 8.4E-06 |
| | | 2020-03-11 | 16440 | 11260 | 372 | 10527 | 11993 | 1.46 | 2.7E-31 |
| | | 2020-03-18 | 13315 | 11716 | 376 | 10975 | 12457 | 1.14 | 3.2E-05 |
| A | Alimentary tract and metabolism | 2020-03-04 | 1725 | 1508 | 46 | 1417 | 1599 | 1.14 | 4.9E-06 |
| | | 2020-03-11 | 2189 | 1512 | 47 | 1419 | 1605 | 1.45 | 1.3E-32 |
| | | 2020-03-18 | 1781 | 1559 | 48 | 1465 | 1653 | 1.14 | 5.7E-06 |
| | | 2020-04-22 | 1459 | 1618 | 46 | 1527 | 1709 | 0.90 | 7.1E-04 |
| | | 2020-04-29 | 1265 | 1423 | 46 | 1331 | 1515 | 0.89 | 8.1E-04 |
| B | Blood and blood forming organs | 2020-03-04 | 1658 | 1432 | 67 | 1300 | 1564 | 1.16 | 8.9E-04 |
| | | 2020-03-11 | 2041 | 1441 | 68 | 1307 | 1576 | 1.42 | 7.2E-16 |
| | | 2020-06-24 | 1286 | 1557 | 67 | 1424 | 1689 | 0.83 | 7.6E-05 |
| | | 2020-12-23 | 1049 | 796 | 67 | 663 | 928 | 1.32 | 2.2E-04 |
| C | Cardiovascular system | 2020-03-04 | 4316 | 3691 | 131 | 3434 | 3949 | 1.17 | 3.3E-06 |
| | | 2020-03-11 | 5753 | 3736 | 133 | 3473 | 3998 | 1.54 | 4.7E-35 |
| | | 2020-03-18 | 4502 | 3869 | 135 | 3604 | 4135 | 1.16 | 4.8E-06 |
| D | Dermatologicals | 2020-03-11 | 16 | 13 | 0.56 | 12 | 15 | 1.19 | 8.4E-06 |
| | | 2020-12-16 | 17 | 15 | 0.56 | 13 | 16 | 1.16 | 7.5E-05 |
| G | Genito urinary system and sex hormones | 2020-03-11 | 944 | 728 | 29 | 670 | 786 | 1.30 | 5.0E-12 |
| H | Systemic hormonal prep, excluding sex hormones | 2020-03-04 | 342 | 280 | 13 | 253 | 306 | 1.22 | 5.1E-06 |
| | | 2020-03-11 | 453 | 280 | 14 | 253 | 307 | 1.62 | 2.2E-27 |
| | | 2020-04-01 | 387 | 296 | 14 | 268 | 325 | 1.30 | 2.0E-09 |
| J | General antiinfectives for systemic use | 2020-03-11 | 103 | 92 | 3.4 | 85 | 99 | 1.13 | 8.6E-04 |
| | | 2020-04-15 | 74 | 87 | 3.4 | 80 | 94 | 0.85 | 2.0E-04 |
| | | 2020-04-22 | 72 | 87 | 3.3 | 80 | 94 | 0.83 | 9.6E-06 |
| | | 2020-04-29 | 64 | 79 | 3.4 | 72 | 85 | 0.82 | 3.2E-05 |
| | | 2020-05-06 | 71 | 87 | 3.3 | 81 | 94 | 0.81 | 2.3E-06 |
| | | 2020-05-13 | 71 | 84 | 3.3 | 78 | 91 | 0.85 | 1.4E-04 |
| | | 2020-05-20 | 63 | 75 | 3.4 | 69 | 82 | 0.84 | 2.9E-04 |
| | | 2020-05-27 | 71 | 85 | 3.3 | 78 | 92 | 0.83 | 3.6E-05 |
| | | 2020-06-10 | 74 | 88 | 3.3 | 82 | 95 | 0.84 | 2.7E-05 |
| | | 2020-06-24 | 76 | 89 | 3.3 | 83 | 96 | 0.85 | 1.1E-04 |
| | | 2020-11-04 | 78 | 89 | 3.3 | 83 | 96 | 0.87 | 4.8E-04 |
| | | 2020-12-09 | 80 | 94 | 3.3 | 88 | 101 | 0.86 | 6.5E-05 |
| | | 2020-12-16 | 84 | 97 | 3.4 | 90 | 103 | 0.87 | 3.1E-04 |

| number of DDD per 1000 | | | | | | | | | |
|---|---|---|---|---|---|---|---|---|---|
| ATC anatomical main group | | Week start | Observed | Predicted | St Error | Lower 95% CL | Upper 95% CL | Ratio (observed/ predicted) | p-value* |
| L | Antineoplastic and immunomodulating agents | 2020-03-04 | 175 | 153 | 5.2 | 142 | 163 | 1.15 | 2.5E-05 |
| | | 2020-03-11 | 215 | 151 | 5.3 | 141 | 161 | 1.43 | 7.1E-26 |
| | | 2020-03-18 | 177 | 152 | 5.3 | 142 | 163 | 1.16 | 5.6E-06 |
| | | 2020-04-29 | 127 | 145 | 5.2 | 135 | 156 | 0.87 | 5.5E-04 |
| M | Musculo-skeletal system | 2020-03-11 | 354 | 268 | 9.8 | 249 | 287 | 1.32 | 1.3E-15 |
| | | 2020-04-22 | 257 | 290 | 9.7 | 271 | 309 | 0.88 | 6.7E-04 |
| N | Nervous system | 2020-03-11 | 2525 | 1957 | 66 | 1826 | 2087 | 1.29 | 2.4E-15 |
| P | Antiparasitic products | 2020-03-04 | 5.6 | 4.3 | 0.36 | 3.5 | 5.0 | 1.32 | 2.5E-04 |
| | | 2020-03-11 | 7.3 | 4.2 | 0.37 | 3.5 | 4.9 | 1.75 | 2.9E-15 |
| | | 2020-03-18 | 9.3 | 4.2 | 0.37 | 3.5 | 4.9 | 2.23 | 4.7E-31 |
| | | 2020-03-25 | 7.7 | 4.3 | 0.39 | 3.6 | 5.1 | 1.79 | 1.1E-15 |
| R | Respiratory system | 2020-03-04 | 1050 | 872 | 47 | 778 | 965 | 1.20 | 2.2E-04 |
| | | 2020-03-11 | 1622 | 927 | 48 | 832 | 1022 | 1.75 | 8.8E-33 |
| | | 2020-03-18 | 1326 | 1043 | 49 | 947 | 1140 | 1.27 | 2.7E-08 |
| | | 2020-03-25 | 1255 | 993 | 51 | 892 | 1094 | 1.26 | 6.9E-07 |
| | | 2020-04-01 | 1213 | 963 | 51 | 863 | 1063 | 1.26 | 1.7E-06 |
| S | Sensory organs | 2020-03-04 | 175 | 150 | 6.8 | 137 | 164 | 1.17 | 2.5E-04 |
| | | 2020-03-11 | 218 | 151 | 6.9 | 138 | 165 | 1.44 | 5.3E-18 |

Abbreviations: Defined Daily Dose (DDD), Confidence Limit (CL), Anatomical Therapeutic Code (ATC)
*p-values are presented uncorrected for multiple comparisons.
