# Supplementary material for: Patterns of prescription dispensation and over-the-counter medication sales in Sweden during the COVID-19 pandemic
Source: PLoS One. 2021 Aug 13;16(8):e0253944. doi: 10.1371/journal.pone.0253944 (PMC8362980; doi:10.1371/journal.pone.0253944)
Supplement: S2 Table — (PDF) [file pone.0253944.s005.pdf]

Supplementary Table 2

Observed versus predicted volume of defined daily doses sold of over-the-counter medications per 1000 inhabitants in Sweden for weeks in 2020 with statistically significant differences

number of DDD per 1000									
ATC anatomical main group		Week start	Observed	Predicted	St Error	Lower 95% CL	Upper 95% CL	Ratio (observed/ predicted)	p-value*
	Any ATC	2020-02-26	1521	1299	64	1172	1426	1.17	7.3E-04
		2020-03-04	1727	1234	64	1107	1361	1.40	8.5E-13
		2020-03-11	2504	1277	66	1148	1407	1.96	1.3E-45
		2020-03-18	2002	1405	66	1274	1536	1.42	2.1E-16
		2020-03-25	1689	1384	70	1247	1521	1.22	1.9E-05
		2020-05-27	1120	1352	65	1225	1480	0.83	4.1E-04
A	Alimentary tract and metabolism	2020-02-26	462	395	17	362	428	1.17	8.2E-05
		2020-03-04	480	377	17	344	410	1.27	4.1E-09
		2020-03-11	617	380	17	347	414	1.62	5.2E-31
		2020-03-18	506	392	17	358	426	1.29	3.8E-10
		2020-03-25	456	393	18	358	429	1.16	6.5E-04
		2020-05-27	290	387	17	354	420	0.75	2.7E-08
		2020-06-24	339	406	17	373	439	0.83	8.2E-05
		2020-08-05	285	356	17	323	389	0.80	3.4E-05
		2020-08-12	256	366	17	333	399	0.70	4.5E-10
		2020-08-19	301	396	17	363	430	0.76	3.9E-08
		2020-08-26	315	402	17	368	435	0.78	5.4E-07
		2020-09-02	301	380	17	347	413	0.79	4.4E-06
		2020-09-09	287	372	17	339	405	0.77	1.0E-06
		2020-09-16	296	382	17	349	415	0.77	6.7E-07
		2020-09-23	346	405	17	372	438	0.85	4.8E-04
		2020-09-30	458	386	17	353	419	1.19	2.5E-05
		2020-10-07	471	366	17	333	399	1.29	2.4E-09
		2020-10-14	452	383	17	350	416	1.18	6.4E-05
B	Blood and blood forming organs	2020-03-11	96	72	4.5	63	81	1.34	2.4E-07
		2020-05-06	56	71	4.5	62	80	0.79	7.6E-04
		2020-05-13	55	71	4.5	63	80	0.76	2.1E-04
		2020-05-27	46	74	4.5	65	83	0.63	4.0E-09
		2020-06-03	36	68	4.5	59	77	0.52	6.4E-12
		2020-06-10	35	76	4.5	67	85	0.46	4.0E-17
		2020-06-17	24	70	4.5	61	79	0.34	3.6E-20
		2020-06-24	23	81	4.5	72	89	0.28	3.3E-28
		2020-11-18	55	78	4.5	70	87	0.70	4.7E-07
		2020-11-25	35	80	4.5	71	89	0.44	1.3E-19
		2020-12-02	27	76	4.5	68	85	0.36	2.7E-22

number of DDD per 1000									
ATC anatomical main group		Week start	Observed	Predicted	St Error	Lower 95% CL	Upper 95% CL	Ratio (observed/ predicted)	p-value*
		2020-12-09	24	78	4.5	69	87	0.31	1.2E-25
		2020-12-16	46	81	4.5	72	90	0.57	3.8E-13
G	Genito urinary system and sex hormones	2020-03-11	49	39	2.8	33	44	1.26	4.5E-04
		2020-05-27	32	41	2.8	35	46	0.77	9.6E-04
M	Musculo-skeletal system	2020-02-26	211	156	5.8	144	167	1.35	6.6E-18
		2020-03-04	286	147	5.8	136	159	1.94	1.5E-60
		2020-03-11	379	145	5.9	133	157	2.61	1.8E-96
		2020-03-25	89	151	6.3	139	164	0.59	4.8E-19
		2020-04-01	102	153	6.2	141	165	0.67	5.2E-14
		2020-04-08	96	121	6.2	109	133	0.79	7.2E-05
		2020-04-15	114	141	5.8	130	153	0.81	7.3E-06
		2020-04-22	106	150	5.8	139	162	0.71	1.2E-12
		2020-04-29	102	135	5.8	124	147	0.75	3.1E-08
		2020-05-06	102	136	5.8	125	148	0.75	1.4E-08
		2020-05-13	103	137	5.8	126	149	0.75	2.1E-08
		2020-05-20	102	134	5.9	122	145	0.77	2.6E-07
		2020-05-27	102	149	5.8	137	160	0.69	1.4E-13
		2020-06-03	86	135	5.8	124	147	0.64	5.0E-15
		2020-06-10	99	151	5.8	139	162	0.66	4.2E-16
		2020-06-17	85	140	5.8	129	152	0.60	4.8E-18
		2020-06-24	114	161	5.8	149	172	0.71	1.0E-13
		2020-07-01	118	149	5.8	137	160	0.79	3.3E-07
		2020-07-08	109	138	5.8	126	149	0.79	9.4E-07
		2020-07-15	110	138	5.8	127	150	0.80	3.1E-06
		2020-08-05	114	134	5.8	122	145	0.85	8.6E-04
		2020-08-12	109	138	5.8	126	149	0.79	1.2E-06
		2020-08-19	122	151	5.8	140	163	0.80	7.0E-07
		2020-08-26	135	156	5.8	145	168	0.86	2.9E-04
		2020-09-09	126	148	5.8	136	159	0.85	2.1E-04
		2020-09-16	123	149	5.8	138	161	0.82	1.0E-05
		2020-09-23	131	159	5.8	148	170	0.83	3.8E-06
		2020-09-30	127	149	5.8	138	161	0.85	1.6E-04
		2020-10-07	122	143	5.8	132	155	0.85	3.1E-04
		2020-10-21	131	160	5.8	148	171	0.82	1.4E-06
		2020-12-09	132	159	5.8	147	170	0.83	6.6E-06
		2020-12-16	148	172	5.9	161	184	0.86	3.9E-05
		2020-12-23	107	136	5.8	124	148	0.78	1.0E-06
N	Nervous system	2020-02-26	220	171	6.9	157	184	1.28	2.3E-11
		2020-03-04	281	162	6.9	149	176	1.73	1.0E-41

number of DDD per 1000									
ATC anatomical main group		Week start	Observed	Predicted	St Error	Lower 95% CL	Upper 95% CL	Ratio (observed/ predicted)	p-value*
		2020-03-11	467	161	7.0	147	175	2.91	2.5E-104
		2020-03-18	399	164	7.1	150	178	2.43	4.3E-83
		2020-03-25	221	166	7.4	151	181	1.33	3.4E-12
		2020-05-27	121	162	6.9	148	175	0.75	1.7E-08
		2020-06-03	121	147	6.9	134	161	0.82	1.9E-04
		2020-06-17	125	151	6.9	137	164	0.83	2.7E-04
R	Respiratory system	2020-03-11	895	480	58	367	594	1.86	1.2E-11
		2020-03-18	821	584	58	469	699	1.41	6.4E-05
		2020-03-25	804	559	61	439	680	1.44	8.3E-05
		2020-06-10	748	551	56	439	662	1.36	5.9E-04

Abbreviation: Defined Daily Dose (DDD), Confidence Limit (CL), Anatomical Therapeutic Code (ATC)
*p-values are presented uncorrected for multiple comparisons.
